# Effect of Metformin For Decreasing Proliferative Marker in Women with Endometrial Cancer: A Randomized Double-Blind Placebo-Controlled Trial

**DOI:** 10.31557/APJCP.2020.21.3.733

**Published:** 2020-03

**Authors:** Kittisak Petchsila, Nisa Prueksaritanond, Putsarat Insin, Marut Yanaranop, Nutpacha Chotikawichean

**Affiliations:** 1 *Department of Obstetrics and Gynecology, *; 2 *Department of Pathology and Laboratory Medicine, Rajavithi Hospital, Bangkok, Thailand. *

**Keywords:** Metformin, endometrial cancer, Ki-67 index, proliferative marker

## Abstract

**Objective:**

To compare the Ki-67 index of endometrial cancer cells before and after treatment between the metformin and placebo group in women with endometrial cancer (EC).

**Methods:**

This study was a randomized, double-blind, placebo-controlled trial conducting in non-diabetic women who diagnosed with endometrioid EC and had a schedule for elective surgical staging at Rajavithi Hospital between August 2018 and June 2019. Tissue specimens were obtained via endometrial curettage at the time of initial diagnosis (pre-treatment) and hysterectomy (post-treatment) to assess the value of the Ki-67 index by immunochemistry. Patients were randomly assigned into 2 groups: metformin and placebo group. Metformin 850 mg or placebo 1 tab were administered once daily for at least 7 days, starting on the first morning after recruitment until one day before surgery. Baseline characteristics (e.g., age, body mass index, co-morbidities) including surgical and pathological characteristics were recorded. The metabolic effect of metformin was also evaluated by a recording of fasting blood sugar, HbA1C and potential adverse events including nausea, vomiting, dizziness, and hypoglycemic symptom.

**Results:**

A total of 49 EC patients were included in this study. Twenty-five patients were assigned to the metformin group and 24 patients were assigned to the placebo group. Baseline demographic, surgical, and pathological characteristics between the 2 groups were similar. Metformin significantly changed the Ki-67 index relative to placebo, with a mean decrease of 23.3% (p=0.001) and a mean proportional decrease of 39.1% (p=0.006) before and after treatment. Additionally, no significant differences were detected in metabolic effects and adverse events between the metformin and the placebo groups.

**Conclusion:**

Short-term treatment with an oral metformin significantly reduced a proliferative marker Ki-67 index in women with endometrioid EC awaiting surgical staging. This study supports the biological effect of metformin in EC and potential applications in the adjuvant treatment in EC patients.

## Introduction

Endometrial cancer (EC) is the most common gynecologic malignant tumor of the female reproductive tract. In 2019, 61,880 new cases and 12,160 deaths related to EC were reported worldwide (Siegel et al., 2019). Compared to other cancers, EC is fairly common, but the number of new cases appears to be on a rising trend and becomes the second rank of female genital tract cancer after breast cancer. The majority of EC cases are diagnosed at an early stage and have a relatively good survival rate with the 5-year survival rate for local diseases of 95%. However, women diagnosed with advanced-stage or recurrence disease have a very poor prognosis with a 5-year survival rate of below 50% (SEER, 2019). 

The increasing incidence of EC has been attributed to various factors, including obesity, diabetes, reproductive factors, and menopausal hormonal therapy that led to unopposed estrogen stimulation which is a principle fundamental for development of EC (Duncan et al., 2012). Currently, obesity and diabetes are increasing at an alarming rate and have been linked with an increased risk of mortality from EC (Fader et al., 2009; Friberg et al., 2007). Women who developed EC with diabetes and obesity will have a decreased life expectancy when compared to non-diabetes and non-obese with same cancer (Calle et al., 2003). Although impaired glucose tolerance and insulin resistance may facilitate the initiation and progression of EC, effective diabetes control has been suggested to prevent EC (Zhang et al., 2010). Therefore, the research of novel therapeutic targets which target glucose metabolism and insulin resistance, such as metformin, as a role of treatment of EC is challenging for combat the increasing EC burden. 

Metformin is an oral biguanide drug which has been recommended as a first-line treatment for type II diabetes and has beneficial effects on various markers of metabolic syndrome (American Diabetes Association, 2019). The association between obesity, diabetes, and EC has led to the hypothesis that metformin may be effective in preventing and treating EC by the main effect in lowering blood glucose concentrations, increasing insulin sensitization, and reducing plasma fasting insulin levels. Additionally, metformin users also show a tendency toward sustained weight loss which is a potential benefit in obesity (Knowler et al., 2009). Many studies suggest that metformin use significantly decreased the incidence and improved survival outcomes of a wide range of cancers, such as breast, lung, liver, pancreatic, colorectal, prostate, cervical, and ovarian cancer, including those with EC (Zhan et al., 2014; Hanprasertpong et al., 2017; Xu et al., 2015). Although the exact mechanism is still not fully understanding, it is hypothesized that metformin’s anti-cancer effects are mediated by indirect and direct effects on tumor growth (Viollet et al., 2012; Pollak et al., 2012). The indirect effects may be the inhibition of liver gluconeogenesis which results in a decrease in both insulin and glucose in the circulating system. While the directs effects are mediated through activation of critical signaling pathways such as adenosine monophosphate-activated protein kinase (AMPK) activation which results in the inactivation of mammalian target of rapamycin (mTOR) pathway. Alteration in the mTOR pathway as well as inactivating PTEN mutations and PIK3CA amplification are common in EC, thus metformin use is believed to have a great benefit in this cancer (Gehrig et al., 2010; Dedes et al., 2011).

Nowadays, several preoperative window studies of metformin in newly diagnosed EC patients planning to undergo surgery have been conducted. All have been a small, open-label, non-randomized trial, and demonstrated with inconsistent results. Three of these studies found that metformin reduced Ki-67 staining in endometrial tumors after metformin treatment while the other found no effect (Schuler et al., 2015; Mitsuhashi et al., 2014; Laskov et al., 2014; Soliman et al., 2016). Although almost studies suggested a potential benefit from metformin treatment in reducing tumor growth and increase apoptosis, the quality of evidence was low because of an inadequate methodology. Therefore, we plan to conduct an adequately powered, placebo-controlled, double-blind, randomized trial in order to determine the effects of oral metformin on the EC cells in women with newly diagnosed EC with the hypothesis that treatment with oral metformin would decrease EC cell growth by evaluating effects on the marker of cell proliferation (Ki-67).

The objective of this study was to compare the proliferative marker (Ki-67) index of EC cells before and after treatment between the pre-operative oral metformin versus placebo in women with newly diagnosed EC waiting for primary surgery, including evaluating metabolic effect before and after metformin treatment in these patients.

## Materials and Methods

This randomized, double-blinded, placebo-controlled trial was conducted at the Department of Obstetrics and Gynecology, Rajavithi Hospital, Bangkok, Thailand, between August 1st, 2018 and June 30th, 2019. This clinical trial was prospectively registered at ClinicalTrial.gov (Clinical trials registration: NCT03618472) and approved by the Institutional Research Committee (IRB) of Rajavithi Hospital.

Women with newly diagnosed EC who had a schedule for elective comprehensive surgical staging were invited to join this study. Eligible women were aged 18 years or older, had histologically confirmed endometrioid EC, and were scheduled to undergo surgical treatment by hysterectomy in the following 30 days. Exclusion criteria included non-endometrioid histologic subtype, prior or concomitant of other cancer within 5 years, Eastern Cooperative Oncology Group (ECOG) performance status >2, medically inoperable, known history of diabetes, pregnancy, currently taking metformin or other hypoglycemic drugs, severe renal impairment (serum creatinine >1.5 mg/dL or eGFR <45 mL/min/1.732 m^2^), hepatic impairment, allergy or any contraindication to biguanides, taking hormonal therapy (e.g., estrogen, progesterone) or chemotherapeutic agents within 3 months, and inability to give informed consent.

*Randomization and Blinding*

After the study was approved, eligible women who gave informed consent were enrolled. All women were treated with the same standard guidelines. The participants were randomly allocated in a 1:1 ratio to either metformin or placebo group. An independent co-investigator generated the randomization sequence by computer-generated software using a block-of-four method. Randomization numbers were stored in sequentially numbered sealed-opaque envelopes. The drugs and placebo were prepared before the study by an independent pharmacist who was not involved in the study. When a study subject met the inclusion criteria, the nurses selected a sequentially numbered sealed-opaque envelopes and assigned the participants to their respective groups. All participants, clinicians, outcome assessors, and investigators were blinded to the treatment assignment throughout the conduct of the study. Blinding was achieved by using a placebo and active drug that were identical in appearance, packaging, labeling, and instruction for use. 

*Study procedures*

At the recruitment period, a medical history was taken, serum renal and liver function determined, and a pregnancy test was performed. All participants, who met the inclusion criteria and willing to participate in this study, provided written informed consent. Baseline characteristics were recorded in the case record form, including height, weight, and body mass index (BMI). Venipuncture was done and serum was obtained to measure fasting blood sugar (FBS), and glycated hemoglobin (HbA1C). The endometrial tissue taking from the fractional and curettage procedure was requested in the form of a formalin-fixed paraffin-embedded block (FFPE) and sent to the Department of Pathology and Laboratory Medicine, Rajavithi Hospital for review and evaluate the expression of Ki-67 staining. Metformin (850 mg per tablet) were assigned to the treatment group and the corresponding placebo (starch-based placebo) to the placebo group. The study drug was prepared in the bottle containing 30 tablets and the drug dose was one tablet taken by mount once daily after the morning meal. Treatment was started on the first morning after the day of recruitment and continued for at least 7 days. All participants should take the study drug until one day before surgery and the availability duration of treatment was approximately 12-30 days. Participants documented treatment compliance and drug adverse events, such as nausea, vomiting, diarrhea, dizziness, hypoglycemic symptom, and anaphylaxis, which supported by every biweekly telephone contact with the research team. Blood glucose was also monitored on Monday each week until the day of admission and provided by the primary health care center. Study drug will be withheld in the case of intolerable side effects and reported to IRB. 

On the day of the scheduled surgery, participants returned unused drugs to nurse and the number of drugs was counted and compared with the document of drug compliance. Repeat checking of the liver and renal function, electrolyte, and FBS before going to the operating room. Then, comprehensive surgical staging operations were performed in the same manner by gynecologic oncologist staff. The operative procedure, operative finding, blood loss, operative time, and complication during and after operation were recorded. An FFPE block was obtained from the hysterectomy specimen for immunochemistry (Ki-67) staining and the results were used for analysis comparing with tissue specimens obtained via endometrial curettage at the time of initial diagnosis. Finally, consultant pathologists assessed all tissue specimens from the surgical procedure for the finalized histopathological diagnosis. The histological subtype, grade, stage, depth of myometrial invasion, the presence of lympho-vascular invasion, lymph node status, and peritoneal fluid/cytology were assessed by using the International Federation of Gynecology and Obstetrics (FIGO) 2009 for endometrial cancer staging system.

*Outcome measurement*

The primary outcome was comparing the percentage of Ki-67 expression measured at the initial diagnosis of EC (baseline) and after treatment. The expression of Ki-67 was associated with the proliferative activity of malignant tumors that was allowing it to be used as a marker of tumor aggressiveness. Additionally, the Ki-67 was also used as a prognostic marker and a potential therapeutic target for cancer diagnosis and treatment because of a strong correlation with patient survival. If the study showed a negative or low percentage of Ki-67 expression, the patients will be a more favorable prognosis (Li et al., 2015).

After the tissue specimen was obtained, all tissue was fixed by 10% buffered formalin and sent to the Department of Pathology and Laboratory Medicine. Formalin-fixed tissue was paraffin-embedded and 3-5 µm sections were cut and put on the slides. These sections were deparaffinized with xylene, rehydrated in a graded series of alcohol and then the slides were stained by using the BenchMark XT automated slide stainers for expression of Ki-67. The primary antibody, Ki-67 antibody MIB-1 clone (DAKO, code M7240), was incubated and then followed by the detection kit. After the end of the run, slides were counterstained with hematoxylin and dehydrated through graded alcohols, cleared with xylene, and mounted slides with permanent mounting medium. Negative and positive controls were also performed for quality assurance.

To compare pre- and post-treatment endometrial tissues among each other, all slides were analyzed by light microscopy (Olympus BX43 light microscope, Tokyo, Japan) and assessed for percentage of distribution of Ki-67 staining (0%-100%). Also, slides were estimated by using a scoring system: low, ≤15% Ki-67 positive cells; intermediated, 16-30% Ki-67 positive cells; and highly proliferative, >30% Ki-67 positive cells (Jonat et al., 2011). All scoring was performed by two independent pathologists (N.C., M.Y.) who were blinded to treatment assignment. There was a 46.9% agreement on the pre-treatment Ki-67 index with a kappa of 0.15 (95%CI; 0.01,0.28) which is a slight agreement range between two observers. For post-treatment Ki-67, there was a 53.1% agreement with a kappa of 0.28 (95%CI; 0.09,0.48) which is fair agreement range between two observers. However, any discrepancies were reviewed together and resolved by consensus agreement resulting in the mean score used for the analysis.

The secondary outcome was comparing tumor grading of post-treatment endometrial cancer cell and the change in serum markers of insulin resistance (FBS) from baseline to the end of treatment between metformin and placebo group. The potential adverse events including nausea, vomiting, diarrhea, dizziness, hypoglycemic symptom, and anaphylaxis, were also assessed along the time of conducting the study. 

*Statistical Analysis*

The sample size calculation was estimated base on the rate of mean Ki-67 expression using the formula for two independent means. The rate of mean Ki-67 expression and standard deviation (SD) in the metformin and control group from Sivalingam’s study were 37.4±20.9% and 58.1±26.2%, respectively (Sivalingam et al., 2016). The sample size of each group was 21 women. With adjustments for a drop-out or withdrawal rate of 10%, a minimum of 24 women in each group was required to detect a statistical difference with 80% power at the 0.05 alpha-level. Therefore, a total of 48 women would be required for this study.

Data from all eligible patients were planned to analyze for the primary endpoint on an intention-to-treat basis but there was one patient denied for surgery. Thus, the primary endpoint analysis was performed in the per-protocol population which consisted of only patients without protocol violations. Categorical variables were expressed as frequency and percentage. Then, a comparison of proportions between groups was performed using a Chi-square or Fisher’s exact test. Continuous variables were expressed as the mean and SD for normally distributed data and were compared between groups using an independent t-test. The nonparametric continuous variables were reported as the median with their interquartile range (IQR) and compared between groups using a Mann-Whiney U test. Changes in the Ki-67 index was compared between the treatment groups using a repeated measure analysis of variance (ANOVA) with treatment group as a fixed effect. All statistical analyses were performed using IBM SPSS Statistics version 23.0 (IBM Cooperation, New York, USA) and a level of p-value less than 0.05 was accepted as being statistically significant.

## Results

**Figure 1:**
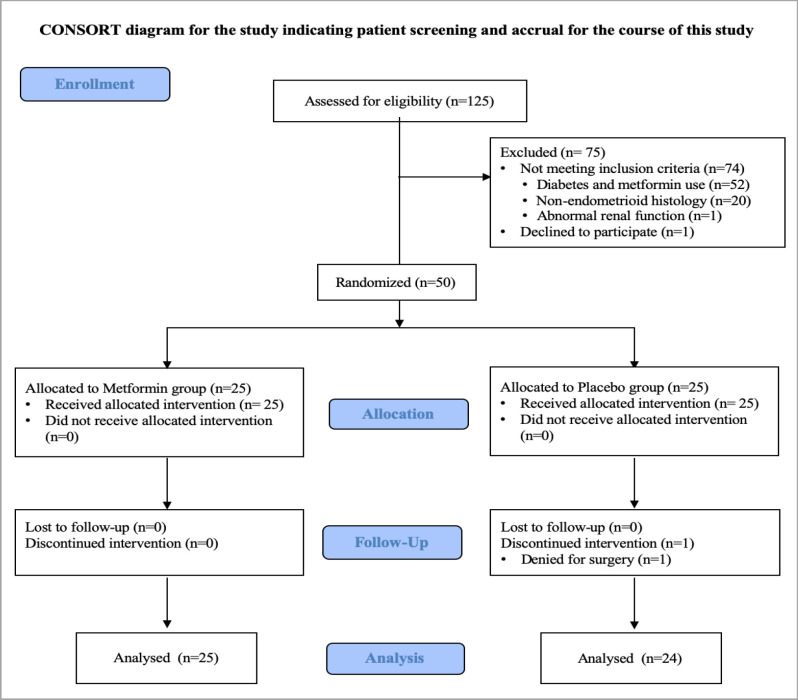
Participant Flow Diagram after Randomization to either Metformin or Placebo Group

From 1st August 2018 to 30th June 2019, a total of 125 EC patients who had been scheduled for elective surgery at Rajavithi Hospital were enrolled and assessed to determine their eligibility for the study as described in Figure 1. Of those patients assessed, 74 patients were excluded primarily because of existing the exclusion criteria: a known history of diabetes and current metformin used (n=52), non-endometrioid histology (n=20), and abnormal renal function (n=2). One patient declined to participate, thus leaving 50 patients were available for the randomization. Because one patient denied for the surgery after the process of randomization, 50 patients made up the per-protocol population with 25 patients in the metformin group and 24 patients in the placebo group. The summary statistics for the demographics and tumor characteristics at baseline are presented in Table 1. The baseline characteristics of the patients were well balanced between the two groups. More than half of all patients (55.1%) were overweight or obese and we found that the patients in the placebo group were overweight or obese slightly higher than the metformin group (66.7% vs 44%, p=0.216). Sixty percent of all patients were postmenopausal women with were evenly matched in mean age (55.5 vs 54.9 years, p=0.839) in the metformin and the placebo group, respectively. For tumor characteristics, most patients undergo laparotomy, had low grade, and early-stage tumors. 

Baseline and post-treatment endpoints compared between the metformin and the placebo group is shown in Table 2. There was no statistical difference in the baseline levels of Ki-67 expression among patients who were assigned to the metformin and the placebo group. The mean baseline levels of Ki-67 expression were slightly higher among patients in the metformin group than patients in the placebo group (mean 52.8% vs 51.8%, p=0.874). After treatment, the expression of Ki-67 was decreased in the hysterectomy specimens in all patients in both two groups. The post-treatment values of Ki-67 expression were statistically significantly lower among patients in the metformin group than among those in the placebo group (29.6% vs 46.2%, p=0.003). We also determined the mean percent change in Ki-67 expression from baseline treatment and we found that Ki-67 expression levels in the metformin group were significantly greatly decreased than the placebo group. The mean decrease of Ki-67 expression was -23.3% and -5.6% for patients in the metformin and the placebo group, respectively (p=0.001). Additionally, the mean proportional decrease of Ki-67 expression, which calculated from the different proportion equation as showed in Table 2, was -39.1% and -3.3% for patients in the metformin and those in the placebo group, respectively (p=0.006). 

**Figure 2:**
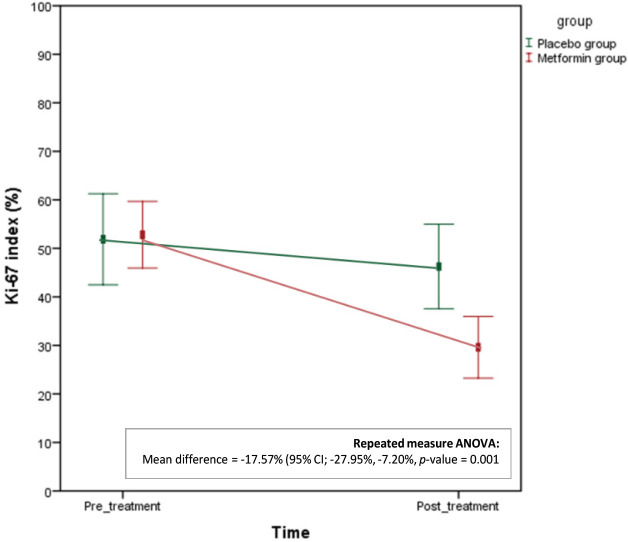
Line Graph Showing the Adjusted Mean Difference in the Ki-67 Index in Pair-interaction of Intervention and Time by Repeated-measure ANOVA Analysis

After adjustment for baseline Ki-67 (pre- and post-treatment) and treatment groups by repeated measure ANOVA analysis, Ki-67 proliferation index was 17.57% lower following metformin treatment (adjusted mean difference -17.57% (95%CI; -27.95%, -7.20%), p= 0.001). The result was showed in Figure 2 that each line represented the adjusted mean difference in the Ki-67 index in pair interaction of intervention and time for the metformin and the placebo group.

With regards to secondary endpoints for this study, there was no statistical difference in the baseline and the change of tumor grade before and after treatment among patients who were assigned to the metformin and the placebo group as shown in Tables 1 and 2.

Table 3 presented the experienced adverse events in the patient who participated in the metformin and the placebo groups. Patients had received metformin for a mean duration of 25.6 days (SD 6.0 days). There was no statistical difference in the change of glucose levels before and after treatment among patients in both two groups (p=0.525). Only 1 of 49 (4%) patients experienced dizziness symptom and this was scored as grade 1 adverse event with could be manageable with a simple treatment. No patients experienced serious adverse events (e.g., hypoglycemia, lactic acidosis, and anaphylaxis) or withdrawal from the study due to the unacceptable adverse event.

**Table 1:** Baseline Patient and Tumor Characteristics Compared between the Metformin and the Placebo Groups

Variables	MFM group		Placebo group		p-value
	(n = 25)		(n = 24)		
Age (years.), mean (SD)	55.5 (10.0)		54.9 (11.9)		0.839
BMI (kg/m^2^), n (%)					0.216
< 18.5	3 (12.0)		0 0.0		
18.5-24.9	11 (44.0)		8 (33.3)		
25-29.9	5 (20.0)		6 (25.0)		
> 30	6 (24.0)		10 (41.7)		
Menopausal status, n (%)					0.686
Premenopausal	8 (32.0)		9 (37.5)		
Postmenopausal	17 (68.0)		15 (62.5)		
Co-morbidity, n (%)					0.281
Hypertension	5 (20.0)		10 (41.6)		
Dyslipidemia	3 (12.0)		2 (8.3)		
Other	1 (4.0)		0 0.0		
Serum marker for insulin resistance					
HbA1C (%), mean (SD)	5.4 (0.4)		5.5 (0.5)		0.522
Duration of drug use (days), mean (SD)	25.6 (6.0)		25.5 (4.9)		0.95
Type of hysterectomy, n (%)					0.463
Laparotomy	22 (88.0)		19 (79.2)		
Laparoscopy	3 (12.0)		5 (20.8)		
Tumor grade from hysterectomy specimen, n (%)					0.764
Grade 1	15 (60.0)		17 (70.8)		
Grade 2	6 (24.0)		5 (20.8)		
Grade 3	4 (16.0)		2 (8.3)		
Tumor size (cm.), mean (SD)	4.3 (1.6)		4 (2.6)		0.584
Myometrial invasion, n (%)					0.144
No invasion	2 (8.0)		0 0.0		
< 50%	13 (52.0)		18 (75.0)		
≤ 50%	10 (40.0)		6 (25.0)		
FIGO 2009 staging, n (%)					0.702
Early stage (I-II)	20 (80.0)		21 (87.5)		
Advanced stage (III-IV)	5 (20.0)		3 (22.5)		
Positive cytology, n (%)	3 (12.0)		0 0.0		0.235
Positive LVSI, n (%)	7 (28.0)		7 (29.2)		0.928

Abbreviations, BMI; Body Mass Index; kg/m^2^, kilograms per meter square; HbA1C, hemoglobin A1C; cm., centimeter; FIGO, International Federation of Gynecology and Obstetrics; SD, standard deviation

**Table 2:** Baseline Details and Post-treatment Endpoints Compared between the Metformin and the Placebo Groups

Variables	MFM group		Placebo group		p-value	p-interaction
	(n = 25)		(n = 24)			
Pre-treatment Ki-67 (%), mean (SD)	52.8 (17.2)		51.8 (15.8)		0.874	
Post-treatment Ki-67 (%), mean (SD)	29.6 (15.8)		46.2 (21.3)		0.003	
Ki-67 index Difference (%), mean (SD)	-23.2 (19.1)		-5.6 (16.8)		0.001	0.001*
Ki-67 index different proportion#	-39.1 (42.1)		-3.3 (45.3)		0.006	
Histology grade difference, n (%)					0.525	
Same grade	17 (68.0)		19 (79.2)			
Decrease grade	5 (20.0)		2 (8.3)			
Increase grade	3 (12.0)		3 (12.5)			

Abbreviations, SD, standard deviation; * p-value interaction of intervention and time from repeated-measure ANOVA analysis; # Difference proportion, [Pre-treatment Ki-67 – Post-treatment Ki-67] / Pre-treatment Ki-67

**Table 3:** Experienced of Adverse Events in Patient who Participated in the Metformin and the Placebo Groups

Variables	MFM group		Placebo group		p-value
	(n = 25)		(n = 24)		
Glucose level (mg/dl), mean (SD)					0/942
Pre-treatment	98.36 (10.6)		96.4 (14.4)		
Post-treatment	98.58 (10.6)		100.04 (12.8)		
Glucose level Difference	-1.96 (13.6)		1.45 (8.0)		0/925
Drug adverse effect					
Dizziness, n (%)					1/000
Mild	1.0 (4.0)		0.0 (0.0)		
Moderate	0.0 (0.0)		0.0 (0.0)		
Severe	0.0 (0.0)		0.0 (0.0)		

Abbreviations, SD, standard deviation; mg/dl, milligram per deciliter

## Discussion

To date, diabetes and obesity are widely acknowledged that these have been associated with increased risk of developing EC and also influenced the severity and the mortality of EC (Friberg et al., 2007; Calle et al., 2003). Therefore, the study of the new pharmacologic agent such as metformin, that modulates insulin sensitivity and probably inhibits tumor cell growth, is become interesting as a role in the adjuvant treatment of EC patients.

EC is an ideal cancer to investigate the pre-operative window study. Pre-treatment samples can be obtained easily during an office endometrial biopsy or curettage and can be compared to endometrial tissue from the hysterectomy after the intervention is given for a while. As above, this model allows for the evaluation of the direct effects of a drug on the EC cells in a relatively short period. Use of metformin in EC may also be the benefit in primary disease prevention and treatment by improving insulin resistance and reducing the incidence of type 2 diabetes which are major risk factors for EC (Viollet et al., 2012; Pollak et al., 2012). Moreover, the low toxicity and the relatively short half-life of metformin make it interesting as a potential adjunctive therapy or even as monotherapy for some EC patients with contraindication to chemotherapy or radiation therapy, including consideration to preserve fertility (Mathur et al., 2008). 

Pre-operative window studies of metformin in patients planning to undergo surgery of EC have shown promising results in metformin’s ability to reduce proliferation index, such as Ki-67, and increase apoptosis (Schuler et al., 2015; Mitsuhashi et al., 2014; Laskov et al., 2014). The Cantrell’s study has also shown that increasing doses of metformin were associated with a decrease in cell proliferation in several EC cell lines (Cantrell et al., 2010). Furthermore, several studies have investigated the effect of metformin on the incidence and risk of EC in women (Tseng et al., 2015; Ko et al., 2015; Luo et al., 2014). However, these studies have presented more conflicting results and it is still unclear whether the use of metformin was associated with a significantly lower risk of EC. Even though a recent meta-analysis found that metformin use was associated with a reduced incidence of EC and could provide better survival outcomes in EC patients. But there are stills the limitations that considerable heterogeneity in factors such as characteristics of patients, indication for metformin use, dosage and duration of metformin use, method of measurement of metformin exposure, and study design (Tang et al., 2017).

This study is a randomized, double-blinded, placebo-controlled trial that evaluated the effect of metformin for decreasing the proliferative marker (Ki-67) index in EC patients. This was among the first study of pre-operative metformin treatment in EC patients without diabetes. We looked at the markers of the Ki-67 proliferation index, which is increasingly being used as a surrogate marker of anti-tumor efficacy in various cancers, to determine the primary endpoint because it could be affected after a short course of oral metformin therapy and previous study has shown that the Ki-67 expression was strong correlation with patient survival (Li et al., 2015).

Consequently, this study provided evidence that the effects of metformin on EC cell proliferation (Ki-67) were significantly decreased among women who received a daily metformin dose of 850 mg administered for approximately 4 weeks before the definitive surgery. We also found that more than half of the patients were overweight or obese that predisposing to undiagnosed type 2 diabetes and insulin resistance. These observations are consistent with the previous study and maybe the reason for showing a positive benefit in pre-operative metformin treatment in EC patients in this study (Fader et al., 2009; Friberg et al., 2007; Calle et al., 2003; Zhang et al., 2010; Kaaks et al., 2002). An alternative explanation is that the restriction only women with endometrioid EC in this study that diabetes and insulin resistance are major risk factors and could be a great benefit from metformin treatment.

In Laskov’s and Sivalingam’s study, they have been described that patients were treated with a higher dose (1,500 mg per day) for a longer period (median 20-36 days) (Laskov et al., 2014). The mean duration of metformin exposure in this study was similar to that of the earlier study but may still be too short. The benefit of a relatively short schedule was the usual time frame for diagnosis to surgical management was not affected and did not interrupt the standard treatment for women who newly diagnosed with EC. In other words, we did not want to delay definitive surgery by exposing patients to longer treatment with metformin. However, the patients in our study were also restricted to receive metformin continue for at least 7 days in order to make sure that the change of Ki-67 index was from the effect of metformin use. 

For the dosage of metformin use, we have chosen a dose of 850 mg per day as the dose of metformin generally in treatment of diabetes in order to prevent the common side effects such as gastrointestinal symptoms which could be made the patients to withdrawal from this study. Moreover, this dosage was also shown the positive effect of metformin in the pre-operative window study (Soilman et al., 2016). 

In our study, we found that metformin as a dose of 850 mg per day has significantly changed the Ki-67 index relative to placebo, with a mean decrease of 23.3% (p=0.001) and a mean proportional decrease of 39.1% (p=0.006) before and after treatment. The two other published studies have reported that the change of Ki-67 index was positive strongly correlated with the average daily dose of metformin received (Sivalingam et al., 2016; Cantrell et al., 2010). It is interesting to presume whether higher doses would have had an even greater impact including proliferative marker, grading, and also clinical endpoints. Such optimal anti-cancer doses of metformin should be further investigated before being used in clinical practice. However, metformin should be commenced at a low dose and build up gradually to limit gastrointestinal adverse events like the standard treatment for treating type 2 diabetes. 

It is interesting to notice that there was no evidence of serious adverse events (e.g., lactic acidosis or anaphylaxis) or withdrawal from the study due to the unacceptable adverse event. Additionally, we also observed that there was no significantly changed in serum biomarkers of insulin resistance (FBS) in EC patients treated with metformin. Therefore, these results assured that metformin has a potential benefit, well-tolerated, and safe for use in EC treatment. Apart from that, inexpensive drugs such as metformin offer an advantage in the pharmaco-economic aspect. This study may facilitate the assessment of a new chemotherapeutic agent in the adjuvant setting for EC treatment, particularly in populations where medical costs represent an important consideration.

There were several strengths in this study. First, this study has a good methodology and well-designed due to a randomized control trial that included a placebo arm, blinding of participants, clinicians, outcome assessors, and investigators. Second, the measurement bias was diminished by using the standard protocol in the specimen preparation process and immunochemistry staining. Moreover, the pathological slides were reviewed by two independent pathologists in order to eliminate the difference observed results from the interpretation of *Ki-67* expression. Although there was a slight and fair agreement, all discrepancies were reviewed together and resolved by consensus agreement. Third, this study provided an intensive protocol for monitoring toxicity from metformin treatment in EC patients to ensure that no serious adverse events were occurring in these patients. Moreover, if any serious adverse event has occurred, prompt management mandatory will be applied.

The main limitations of this study were the small sample size involving a limited number of patients, so caution is required in interpreting the data. The larger randomized trial may be needed before applying in clinical practice guidelines for EC management. However, it still reaches an adequate power although the small sample size. Another limitation of the present study was the reliance on surrogate biomarkers of response *(Ki-67* expression) rather than clinical endpoints such as shrinkage of tumor size or cancer-specific survival. Because of the study design, this study was a pre-operative window study that aims to utilize the treatment period between the initial diagnosis and surgical treatment to screen for potential therapeutic efficacy more rapidly including to avoid compromising patient care which could be affected for the outcome. Further longitudinal studies are required to establish Ki-67 expression as a predictive and prognostic biomarker of long-term clinical outcomes, such as cancer-specific and recurrence-free survival, in EC patients. However, a study for assessing the impact of metformin treatment and survival outcomes in women with EC is underway in the follow-up phase and data from this study are enthusiastically awaited. 

In conclusion, the present study confirmed that short-term pre-operative treatment with an oral metformin dose of 850 mg/day significantly reduced a proliferative marker Ki-67 index in women with endometrioid EC. This evidence was supported the biological effect of metformin in EC and it should be implemented in the primary, adjuvant, neoadjuvant, and advanced disease settings in EC management strategy. 
